# Decision Support Modeling: Data Assimilation, Uncertainty Quantification, and Strategic Abstraction

**DOI:** 10.1111/gwat.12969

**Published:** 2019-12-30

**Authors:** John Doherty, Catherine Moore

**Affiliations:** ^1^ CSIRO Land and Water Floreat WA Australia

## Abstract

We present a framework for design and deployment of decision support modeling based on metrics which have their roots in the scientific method. Application of these metrics to decision support modeling requires recognition of the importance of data assimilation and predictive uncertainty quantification in this type of modeling. The difficulties of implementing these procedures depend on the relationship between data that is available for assimilation and the nature of the prediction(s) that a decision support model is required to make. Three different data/prediction contexts are identified. Unfortunately, groundwater modeling is generally aligned with the most difficult of these. It is suggested that these difficulties can generally be ameliorated through appropriate model design. This design requires strategic abstraction of parameters and processes in a way that is optimal for the making of one particular prediction but is not necessarily optimal for the making of another. It is further suggested that the focus of decision support modeling should be on the ability of a model to provide receptacles for decision‐pertinent information rather than on its purported ability to simulate environmental processes. While models are compromised in both of these roles, this view makes it clear that simulation should serve data assimilation and not the other way around. Data assimilation enables the uncertainties of decision‐critical model predictions to be quantified and maybe reduced. Decision support modeling requires this.

## Abstract 2

*Article Impact Statement*: A conceptual framework that assists modelers in selecting appropriate complexity for decision‐support modeling is presented.

## Introduction

Groundwater models are built on a regular basis to support the making of important decisions. In many cases, the premise of their construction and deployment is that natural processes can be replicated using partial differential equations, and that natural systems can be simulated numerically once calibration has bestowed values on coefficients that appear in those equations values that are “correct” for a certain project site. Once a model has been built and calibrated in accordance with these precepts, it is assumed that the model can then be reliably used to assess the future of a system under a variety of proposed management scenarios.

Anyone who has built a decision support model knows that this characterization of decision support modeling falls far short of the truth. However, those who sell models, and those who pay for them, are rarely so aware. Despite the commissioning of guidance documents such as Barnett et al. (2012) and Joseph et al. (2016) whose purpose is to address a palpable sense of unease in the groundwater industry that models are not living up to expectations, models continue to be produced according to a philosophy that too frequently has failure as its inevitable consequence.

The authors contend that the growing sense of suspicion with which modeling is viewed results from a lack of intellectual rigor in our industry's current approach to decision support modeling. The intention of the present paper is to suggest a more useful philosophical framework for this type of modeling than that which prevails at present. In doing this we draw inspiration from ideas that were originally outlined by Freeze et al. (1990), and from insights provided by the mathematics of parameter estimation and uncertainty analysis. Without an appropriate framework, there can be no metrics by which decision support modeling can be judged. Nor can there be a context for making the important choices that attend construction of a decision support model.

Many of these choices pertain to how complex a model should be in order to serve its decision support role. Despite some cogent reminders of the benefits of simplicity (see, e.g., Hill 2006; Haitjema 2006; Olsthoon 2007), at the present time our industry tends to judge model complexity less harshly than it judges model simplicity. Presumably, this is because a complicated model looks more like “the real thing” than a model that represents reality in a more abstract way. It is the authors' contention that the credibility of decision support modeling has suffered because of this bias. However, in the absence of scientifically based metrics for decision support modeling utility, such a bias is understandable.

**Figure 1:**
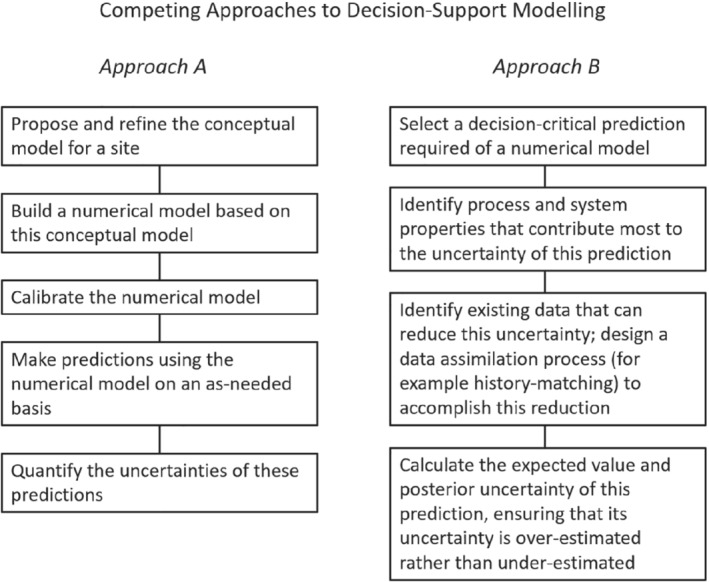
Approach A summarizes the traditional trajectory of decision support modeling; approach B is recommended herein.

As a reference point for the discussion that follows, we provide Figure 1. This figure juxtaposes two different approaches to decision support modeling; these are labeled “approach A” and “approach B” in the figure. We submit that approach A summarizes (albeit in a simplified manner) current beliefs in how decision support modeling should be undertaken; however, we recommend approach B. While differences between the two approaches may be construed as matters of emphasis only, we believe that these differences constitute competing philosophies that have very different modeling outcomes.

## Metrics

In this section, we provide metrics for decision support modeling; these were originally proposed by Doherty and Simmons (2013).

By definition, decision support modeling is undertaken to assist the making of a decision. Presumably, such assistance reduces the chances that a decision will be wrong, at the same time as it estimates those chances. A wrong decision promulgates a course of management action that leads to an unwanted environmental or economic outcome. For ease of expression, we refer to this unwanted management outcome as a “bad thing” in the discussion that follows. Examples of bad things include excessive diminution of spring flow as a result of groundwater extraction, failure of the wall of an open cut mine, and lack of sustainability of groundwater‐sourced irrigation. In some decision contexts, a bad thing may be more nuanced than these. A proposed project may have been given license to proceed on the basis that an unwanted outcome is possible, but can be circumvented if appropriate remedial action is taken if certain monitoring thresholds are crossed. The bad thing in this context is that the triggered remedial action does not prevent the occurrence of the unwanted management outcome.

Regardless of how it is defined, the bad thing must be identified, and its avoidance adopted as a management goal, if its occurrence is to be forestalled.

The occurrence of a bad thing under a proposed course of management action can be posed as a hypothesis. Numerical modeling can then be used to test this hypothesis. In doing so it implements the scientific method. Fundamental to this method is the precept that a hypothesis cannot be accepted; it can only be rejected. In environmental management a bad thing hypothesis can be rejected if it is demonstrably incompatible with any or all of the following:

- The processes that operate within a system,
- The properties of the system,
- The historical behavior of the system.

A history‐matched numerical model incorporates all of these, to some degree at least. In doing so, it provides the receptacles for information on which basis a decision‐pertinent hypothesis may be rejected. This, in the authors' view, is a far more productive way to view a decision support model than as a simulator of natural processes. This is not just a matter of semantics; it has important consequences for how groundwater models are built, history‐matched and deployed. It also provides a framework for addressing the important issue of appropriate model complexity, for this framework can accommodate the fact that a numerical model's capacity to encapsulate the above three hypothesis‐rejection criteria is always compromised.

Compromises are an outcome of the challenges that the modeling of subsurface flow and transport faces. These challenges include the following:

- Subsurface processes are more heterogeneous and more complex than can be represented by a model.
- Even at well‐characterized sites, the three‐dimensional disposition of subsurface materials can be represented only in a stochastic sense.
- These materials have properties that are anisotropic, heterogeneous and variable over orders of magnitude.
- While history matching may reduce the uncertainties of spatially averaged subsurface properties to some degree, the nature of the averaging process is only vaguely known.
- Knowledge of boundary conditions and stresses that drive movement of water and solutes through the subsurface is often only approximate.

In light of the above, the premise that a groundwater model can make an even semi‐accurate prediction of future system behavior, and that this prediction can then provide a basis for decision support, is tenuous. Fortunately, recently developed mathematical and numerical tools allow us to establish for ourselves the extent to which a model's prediction is reliable. Using these tools we can test whether or not a bad thing hypothesis can be rejected. If it can be demonstrated that current data availability does not allow its rejection (at a certain level of confidence), this has important consequences for environmental management, as well as for acquisition of further data.

An immediate consequence of adoption of the scientific method as a conceptual basis for decision support modeling is that it provides a metric for failure. Failure is the occurrence of a statistical error whereby a hypothesis is falsely rejected. This occurs where a modeler declares that a bad thing cannot happen when, in fact, it can.

Other consequences follow. The hypothesis testing imperative demands that predictions made by a model be accompanied by assessments of their uncertainties. To avoid decision support failure, these assessments must be conservative. Where a simple model is deployed (a subject to which we will shortly return), the provision of such a guarantee may be difficult; it may require that an “engineering safety margin” accompany a decision‐critical model prediction. However, without a guarantee of uncertainty conservatism, together with a justification for this guarantee, a modeler cannot provide decision stakeholders with an assurance that his/her modeling has not failed them. Of course, the engineering safety margin may be so wide as to preclude the rejection of any bad thing hypotheses in spite of the fact that information may be available to do so. While such modeling does not constitute failure according to the above definition, it can be characterized as of limited use, or even “useless.” This may thus provide justification for greater modeling complexity, as a more complex model provides more receptacles for information against which a hypothesis can be tested, and possibly rejected. The more complex model does this through facilitating the expression of expert knowledge. At the same time, it may support a level of parameterization detail that enables a better fit to be attained with a calibration dataset. The superior ability of a more complex model to express decision‐relevant information in these ways may promulgate a reduction in the assessed uncertainty of a decision‐relevant prediction. Complexity also endows the model with the ability to actually quantify this uncertainty. This is because a complex model can represent more details of prediction‐salient hydraulic processes and properties than a simple model can; this endows it with the ability to explore the repercussions of less‐than‐full knowledge of these details. The need to ascribe a conservative predictive safety margin to a model prediction is thereby alleviated.

Unfortunately, a trajectory toward increasing modeling complexity soon encounters practical difficulties that force a modeler to make some uncomfortable choices. Incorporation of decision‐pertinent site information, and information forthcoming from historical measurements of system states and fluxes, into a model's structure and parameters (a process for which we use the generic term “data assimilation”), and quantification of the uncertainties associated with a model's predictions, are numerically intensive tasks. They require the use of a model in partnership with specialist software packages that are designed to carry out these tasks. To accomplish these tasks, these packages must run a model many times. Complex models take a long time to run. They also possess a greater propensity for numerical instability than do simple models. A decision support strategy that rests on a complex model may thus be incapable of delivering the promises that model complexity can offer. Suppose that a complex model cannot be used in conjunction with partner software:

- To assimilate information in order to represent that which is known, while
- Quantifying post‐assimilation uncertainties of decision‐critical model predictions by simultaneously representing (in a stochastic sense) that which is unknown.

Then avoidance of modeling failure (as defined above) demands that predictive uncertainties be inflated. As far as supporting a decision is concerned, the complex model then becomes no more useful than a simple model. The expense of its construction and its passing resemblance to “reality” do not constitute valid arguments that it provides a superior basis for decision support than does a simpler model.

In summary, the demands of decision support modeling require that a modeler place him/herself on a spectrum, the two endpoints of which are failure on the one hand and uselessness on the other hand. Unfortunately, this spectrum does not include “success.” This is because models are imperfect, and attempts to seek simulation perfection lead to extremely complex models which are incapable of quantifying and reducing the uncertainties of predictions of management interest. Hence compromises must be sought, some of which are discussed below. In making these compromises, the reference point must always be the decision(s) which the model is required to support.

## Prediction Specificity

The above discussion suggests that modeling serves the decision‐making process best when it is designed to test a specific, decision‐specific hypothesis. This, in turn, suggests that a model should be designed in such a way as to optimize its ability to reduce and quantify the uncertainty of a single prediction. Although other authors have recommended that the prediction that a model is required to make should exert a strong influence on its design (see, e.g., Ferré 2017; Guthke 2017; White 2017), prediction specificity of model design stands in stark contrast to current modeling practice wherein a model is first built to simulate a system, and then tasked with making whatever predictions are required to manage that system. In contrast, prediction specificity of a model's design allows that design to meet the requirements of the hypothesis testing process by providing a context in which the costs and benefits of model complexity can be assessed. In particular, it can accommodate the fact that greater complexity provides more receptacles for decision‐specific information while possibly hindering the passage of that information to those receptacles because of excessive model run times and/or a penchant for numerical instability.

Acceptance of the hypothesis testing imperative of decision support modeling also calls into question the notion that a model should be a “deliverable,” or even that “model” should be a noun and not a verb. It suggests that modeling should be an activity undertaken by scientists to implement the scientific method in relation to the occurrence of a particular bad thing. The “deliverable” is then the outcome of the pertinent hypothesis testing procedure. Where the search for an optimal management strategy requires that a number of hypotheses be tested, then a different model may be needed to test each one of them.

## Data Assimilation and Implications for Model Design

Data assimilation assumes a more important role in groundwater modeling than it does in many other modeling contexts. This is because groundwater flows through an invisible, heterogeneous medium whose properties can be measured only at scales that are very different from that of the system that must be managed. To some extent, estimates of aerially‐averaged properties can be back‐calculated from historical measurements of system states and fluxes. However, large uncertainties in these properties, and in predictions of system behavior that are sensitive to them, remain after these data have been assimilated.

Under the simplifying assumption of linear model behavior, Moore and Doherty (2005) explored what history matching can and cannot achieve. (We employ the term “history matching” interchangeably with “model calibration” in this article, even though they are not quite the same thing.) For a linear model, the relationship between model parameters and model‐calculated counterparts to measurements comprising a calibration dataset can be depicted as a matrix. This is often referred to as the Jacobian matrix. By undertaking singular value decomposition of this matrix, parameter space can be subdivided into two orthogonal subspaces. One of these subspaces is referred to as the “null space.” This space comprises combinations of parameters (i.e., the outcomes of summation and differencing operations applied over many parameters) that are not informed by the calibration dataset. These parameter combinations have as much uncertainty after history matching as they did before history matching. They thus retain their prior uncertainties. These are their uncertainties based on knowledge and data that were available prior to the history matching process through site characterization studies, including point measurements of system properties and aquifer pumping tests.

We refer to the orthogonal complement of the null space as the “solution space.” This space comprises combinations of parameters (often complex spatial averages of parameters) that are uniquely estimable through the history matching process. However, “unique” does not mean “certain,” for estimates of values for these parameter combinations are contaminated by noise associated with the measurements from which they were calculated.

The value of a prediction made by a linear model, as well as the uncertainty of that prediction, can be calculated from the sensitivity of that prediction to a model's parameters; this analysis is sometimes referred to as first‐order second moment analysis. The post‐calibration (i.e., posterior) uncertainty of a prediction is determined by:

- The parameters to which it is sensitive,
- The variability of these parameters as described by their joint prior probability distribution,
- The extent to which their prior variability is constrained through history matching.

Moore and Doherty (2005) partition a prediction's parameter sensitivity into null space sensitivity and solution space sensitivity components. Contributions to its posterior uncertainty can be similarly partitioned. This partitioning has important consequences for the design of a decision support model.

Firstly, suppose that a prediction is sensitive only to solution space parameter components. Then its posterior uncertainty is likely to be considerably smaller than its prior uncertainty. Furthermore, its posterior uncertainty is almost entirely a function of model‐to‐measurement misfit (generally attributed to “measurement noise,” but often with substantial contributions from so‐called “structural noise” arising from model defects). Reduction and quantification of the uncertainty of this prediction therefore requires only that a model be complex enough, and be endowed with enough parameters, to fit the calibration dataset well. As Doherty and Welter (2010) and White et al. (2014) show, a model may need to bear only a passing resemblance to the real‐world to achieve this. Furthermore, its parameters may bear only a loose relationship with spatially averaged system properties—a relationship that may prevent them from being easily informed by field measurements of these properties. This is the case for lumped parameter rainfall‐runoff models; it is even more the case for model emulators that are sometimes used in place of numerical simulators for history matching and decision optimization. Groundwater models which are used to set yearly water allocations, although more “physically based” than rainfall‐runoff models, also fall into this category if historical pumping rates are well known and if the model has been calibrated against an extensive dataset of historical borehole water levels.

It follows from the above considerations that a model which is built to make a decision‐critical prediction which is entirely solution space dependent needs to include only enough process and parameterization complexity as that which is necessary to replicate the past behavior of the system. Furthermore, under these circumstances, the need for prediction specificity of model design may be somewhat relaxed, as the same history matched model can be used to make any prediction that is solution space dependent. The level of fit attained with the calibration dataset is used to statistically characterize the “noise” that is associated with that dataset. This is then used to ascribe uncertainties to solution space‐dependent predictions.

An important design specification for a model that is required to make a solution space dependent prediction is that its parameters be calibration‐adjustable. This requires that the relationships between model outputs and model parameters be continuous (and preferably continuously differentiable). This may require some abstraction in representation of aerially‐averaged processes, and in definition of parameters associated with these processes; see Kavetski and Kuczera (2007) for a discussion of this issue.

Now consider a prediction that is sensitive only to combinations of parameters that lie within the calibration null space. This prediction is sensitive to process and parametric detail that is beyond the capacity of the calibration process to inform. The uncertainty of such a prediction is reduced very little, if at all, through history matching. The design of a model which is optimized to make this kind of prediction does not therefore include the requirement that it be calibrated. This is replaced by the requirement that it be capable of expressing expert geological and other knowledge, taking into account the stochastic nature of this knowledge, particularly as it pertains to system detail. Quantification of the uncertainty of a null space dependent prediction may therefore require that many model runs be undertaken, each based on a different geostatistical realization of system lithologies and properties. Fortunately, the model run burden required for quantification of the uncertainty of such a prediction is generally lighter than that required for history matching. A model which is required to make a null space dependent prediction can therefore be relatively complex, highly physically based, and endowed with a “geologically realistic” representation of the subsurface (possibly involving discrete features such as faults, fractures, alluvial channels and other manifestations of connected (im)permeability), even if the geometries and properties of these features are not readily calibration‐adjustable. In fact, it is a design requirement that hydraulic property heterogeneity that is represented in such a model bear a relationship with reality that allows it to be informed by expert knowledge and by direct measurements of system properties.

It is of interest to note that while the making of null space dependent predictions affords greater tolerance of model complexity, it may also provide opportunities for abstraction/simplification, especially where the hypothesis testing imperative of decision support modeling does not require that the entirety of a predictive probability distribution be characterized. In some decision support contexts, it may be possible to reject a bad thing hypothesis on the basis of worst‐case scenario analysis in which only one extreme of a predictive probability distribution is explored. A model which is built for this exploration can therefore be endowed with parameter values that are purposefully high or purposefully low (this depending on the prediction), over areas of the model domain which are likely to be in excess of those over which connectedness of high/low permeability exists in practice. If a bad thing hypothesis can be rejected under these demonstrably pessimistic circumstances, then greater modeling complexity is not required.

## Blending Data from Different Sources

Unfortunately, the two predictive end members discussed above are not encountered very often when undertaking groundwater modeling for decision support. Most predictions required by most groundwater models are partly solution space dependent and partly null space dependent. Most groundwater models are developed because a significant change is about to be imposed on a system. Because the new stresses to which the system will be subjected are different from those which prevailed in the past, predictions of its response to these new stresses are likely to be sensitive to combinations of parameters that are only partly informed by its response to previous stresses. Reduction and quantification of predictive uncertainty is particularly difficult under these circumstances.

Where a prediction of interest is partly null space dependent and partly solution space dependent then history matching, by definition, constitutes an ill‐posed inverse problem if a model is designed to include all parameters that are required for calculation of the uncertainty of that prediction. If gradient methods, accompanied by mathematical regularization, are employed for solution of this inverse problem, model parameter fields must satisfy certain continuity and smoothness requirements. If ensembles (see below) are used instead, a multiGaussian assumption for prior parameter probability distributions (possibly after transformation) must be invoked. Both of these approaches to history matching imply simplifications of “real” geology, and of its associated spatial variability. The latter can sometimes be expressed using complex categorical parameter fields through methodologies such as multiple‐point geostatistics. (Categorical parameter fields separate lithologies by sharp, pervasive, and often tortuous boundaries whose positions are discontinuous from realization to realization.) Unfortunately, however, the spatial details of categorical parameter fields are difficult to adjust through history matching. Hence groundwater models generally employ somewhat abstract parameter fields instead. Prior probability distributions ascribed to these parameter fields are often equally abstract. A multiGaussian assumption is often invoked; spatial correlation lengths are often guessed.

While this approach to model parameterization may satisfy the demands of the history matching process, it may not satisfy another requirement of decision support modeling, namely that the credibility (or lack thereof) of a parameter field constitute a partial basis for predictive hypothesis testing. Assessment of the uncertainty of a null space dependent prediction requires that a model's parameter field include parameterization detail to which that prediction may be sensitive, regardless of the estimability of this detail. In fact, it is its very inestimability (and hence its occupancy of the calibration null space) which demands its representation. The credibility of a prediction is lowered where its occurrence requires that prediction‐sensitive parameter combinations adopt unrealistic values. If these parameter combinations occupy the null space, they are mathematically unconstrained by history matching. Expert knowledge, encapsulated in the prior parameter probability distribution, therefore comprises their only constraint.

It follows that the prior probability distribution attributed to the somewhat abstract parameter field employed by a groundwater model is of great importance where a prediction required of this model is partly solution space dependent and partly null space dependent. This probability distribution must support sufficient variability and connectivity of (im)permeable heterogeneity to preclude unwarranted rejection of a viable hypotheses. This, in turn, requires parametric representation in the model of any heterogeneity to which the pertinent prediction may be sensitive even if this representation is somewhat abstract. This strengthens the case for prediction specificity of model design, for if a model is required to make many decision‐critical predictions while guaranteeing that it does not underestimate the uncertainty of any of them, its parameterization requirements become excessive.

## Strategic Abstraction in Model Design

In continuing our discussion of decision support modeling, we focus on the data assimilation context within which most groundwater modeling takes place. As stated above, this is the context wherein a prediction of management interest is sensitive to both null and solution space components of a model's parameterization. This context requires that the model be endowed with a readily adjustable parameter field so that it can assimilate information that is resident in a calibration dataset. It also requires that prediction‐sensitive inestimable components of the parameter field be represented with sufficient parameterization density to guarantee their null space occupancy. Furthermore, the prior probability distribution ascribed to these parameter components must allow sufficient variability and connectivity to avoid erroneous preclusion of valid predictive possibilities.

These requirements are not as easily met as may at first appear. Generally, a modeler does not know in advance of the history matching process how this process will subdivide parameter space into its solution and null subspaces. Indeed, because these spaces are spanned by combinations of parameters rather than by individual parameters, details of this partitioning are rarely obvious. All that a modeler can do is to err on the side of predictive uncertainty conservatism. Following this principle, he/she must endow a model with enough parameters to obtain a good fit with the calibration dataset, and more than enough parameters to guarantee that post‐calibration variability of a prediction of management interest is not artificially limited by parameter insufficiency. At the same time, he/she must ascribe to somewhat abstract parameter fields prior probability distributions that encapsulate sufficient variability and potential connectivity of permeable or impermeable material to enable all realistic possibilities for the prediction of management interest whose uncertainty the model is designed to assess.

Practical requirements must also be met. The imperatives of history matching and post history matching predictive hypothesis testing (which, as will be discussed below, can be undertaken in a variety of ways) require that a model's run time be reasonably low, and that the model be numerically stable. The meeting of these requirements impacts its design, its parameterization, and the way in which history matching is performed. Reductions in model run time may be achieved through any of the following means (and many more):

- Coarse vertical and/or horizontal discretization,
- Use of a local model whose boundaries provide an abstract connection with a wider groundwater system,
- Simplified representation of recharge,
- Simplified representation of interaction between ground and surface waters,
- History matching under assumed steady‐state conditions.

Doherty and Christensen (2011) and White et al. (2014) suggest that model abstraction/simplification can be likened to the fixing of certain parameters at non‐adjustable values. These parameters pertain to processes and complexities that are present in the real world but that are absent from the simplified model; we refer to them as “omitted parameters” in the discussion that follows. By including these omitted parameters in formulations of post‐calibration predictive error variance, these authors demonstrate that model abstraction can affect data assimilation and predictive hypothesis testing in the following ways:

- The fit between a calibration dataset and corresponding model outputs may be compromised.
- Calibration‐adjusted parameters may adopt values that compensate for erroneous values attributed to omitted parameters; this may bias some model predictions. Unfortunately, predictions with some degree of null space dependency (in the real world but possibly not in the reduced null space of a simplified model) are particularly prone to calibration‐induced bias.
- Predictions that are entirely solution space dependent (where solution/null space subdivision pertains to the real world and not to the model), do not incur bias in this way, even though some of the parameters of the simplified model to which they are sensitive may themselves incur bias.

Accommodation of calibration misfit incurred by model defects that comprises the first of the above costs has received considerable attention in the modeling literature. See, for example, Kennedy and O'Hagan (2001) and Oliver and Alfonzo (2018) who discuss the importance of addressing the correlation structure of defect‐induced model‐to‐measurement misfit when estimating values for model parameters and when quantifying parameter and predictive uncertainty. White et al. (2014) demonstrate that this issue can often be addressed by careful formulation of the objective function whose value is minimized through the history matching process. By judiciously processing field measurements and corresponding model outputs before matching them (often by subjecting them to lateral, vertical and/or temporal differencing), the effects of model defects on model outputs can often be at least partially “filtered out.” While this type of processing may remove some information from the calibration dataset, the benefits can often outweigh the costs, as the “structural noise” that contaminates the foregone information would have rendered the assessment of predictive uncertainty difficult. Predictive uncertainty may consequentially rise, but is quantifiable.

The second of the above costs is invisible to the history matching process. It is also prediction‐specific, as it will often be possible to construct a simplified model in such a way as to render abstractions in its boundary conditions, and in other aspects of its design, innocuous to one prediction, but not necessarily to another. For example, it may be possible to ameliorate calibration‐induced bias for one particular prediction by allowing parameters that are associated with certain model boundary conditions to be adjustable rather than fixed, and by assigning them generous prior probability distributions in order to guarantee predictive uncertainty conservatism. (Generosity of their prior distributions reflects limitations in the ability of expert knowledge to fully inform them, possibly because of their somewhat abstract nature.) The importance of prediction specificity in model design is again apparent.

We conclude this section by noting that numerical expediency and prediction specificity are not the only factors that should be considered in placing limits on the complexity of a decision support model. The human factor is just as important. Notwithstanding its numerical complexity, even the most sophisticated simulator is replete with abstractions, any one of which may induce bias in one of the many decision‐critical predictions that it may be asked to make. When a model of such complexity is then deployed to test a particular prediction‐pertinent hypothesis, it becomes very difficult for a modeler to guarantee the avoidance of a false rejection of that hypothesis. Furthermore, instead of turning his/her attention to satisfying the metrics of decision support modeling set out above (i.e., avoidance of failure on the one hand and avoidance of uselessness on the other hand), the builder of such a model can become obsessed with meeting other, more nebulous, metrics which are based on an apparent resemblance of a model to reality and on the illusive pursuit of accuracy for a plethora of unstated predictions. A model that is built according to these metrics rapidly becomes too big for a modeler to “get his/her head around.” The task of constructing a simulator whose philosophical basis eschews abstraction rather than embracing it then becomes one of damage minimization as its many defects are hidden in ad‐hoc ways from the critical eyes of reviewers and stakeholders. With every such alteration, the ability of the model to support environmental management according to the metrics stated above slips from the modeler's grasp.

## Testing Predictive Hypotheses

Conceptually, if the occurrence of an unwanted event falls outside the range of uncertainty that is attributed to the corresponding model prediction, the hypothesis that this event will occur can be rejected.

The post‐calibration uncertainty of a prediction made by a highly parameterized model can be explored in a number of ways. These include linear analysis (James et al. 2009; Dausman et al. 2010), linear‐assisted methods such as null space Monte Carlo (Tonkin and Doherty 2009; Doherty 2015), ensemble methods (Chen and Oliver 2013; White 2018) and data space inversion (Satija and Caers 2015; Sun and Durlofsky 2017, 2019). All of these methods are replete with assumptions and limitations. These are associated with (but are not limited to):

- The type of parameterization device with which a model is endowed (for example zones of assumed piecewise constancy or pilot points),
- The number of parameters with which a model is endowed,
- The joint prior probability distribution ascribed to model parameters,
- The degree of connected (im)permeability that this prior probability distribution allows,
- The degree of connected (im)permeability that can be maintained as parameters are adjusted through the history‐matching process.

The last two of these are of particular importance. The geological materials through which groundwater flows are often characterized by long‐range connectedness of high or low permeability material (Fogg 1986; Anderson 1989; Renard and Allard 2013). The complex relationship between connectivity and permeability is difficult to specify in a prior probability distribution ascribed to an upscaled, calibration‐adjustable parameter field. It is also difficult to maintain in the history‐matched parameter field, even where the inverse problem of model calibration is highly ill‐posed. Failure to preserve permeability connectedness may lead to under‐estimation of predictive uncertainty, and hence to false rejection of a bad thing hypothesis.

In some modeling circumstances it may be possible to overcome this problem by granting parameters the flexibility that they need to support the occurrence of a hypothesized bad thing as part of the hypothesis testing procedure itself. We refer to this mode of model usage as “direct predictive hypothesis testing.” It is accomplished in two steps. First, a model is history matched to the calibration dataset using highly parameterized inversion supported by appropriate Tikhonov regularization (i.e., a regularization strategy that achieves parameter uniqueness through specifying that departures from homogeneity be minimized). A modeler learns through this process the amount of model‐to‐measurement misfit that accompanies the use of his/her model. (If this is too great, the model can, of course, be re‐configured to reduce this misfit.) The modeler also learns something about the variance and spatial variability of the upscaled parameter field required of the calibrated model. To some extent, this will reflect the heterogeneity of the real world; however, some of it may be incurred through history matching as parameters adopt values and spatial patterns that compensate for model defects. (If the latter is judged to occur to an undue extent, the modeler may reconfigure his/her model to reduce this effect.)

The model is then re‐calibrated. However, on this occasion the hypothesized bad thing is included in the calibration dataset. That is, the model is asked to match the value of a hypothesized decision‐pertinent prediction; at the same time, it is also asked to maintain its match with the calibration dataset according to misfit criteria which the modeler accepted during the previous calibration exercise. As an outcome of this procedure, the bad thing hypothesis can be rejected if either of the following conditions arise:

- The fitting of the hypothesized prediction results in an unacceptable misfit with the calibration dataset.
- The fitting of the hypothesized prediction requires the introduction into the calibrated parameter field of unrealistic parameter values, or of unrealistic patterns of spatial heterogeneity.

The principle advantage of direct predictive hypothesis testing is that it allows a modeler to select a hypothesis rejection threshold at the same time as the hypothesis is actually being tested. Hence it does not place excessive reliance on predefined stochastic descriptions of either model‐to‐measurement misfit or of spatial parameter variability. If a model is endowed with a sufficiency of prediction‐sensitive parameters (which, as has already been stated, is a prerequisite for its use in decision support), then the direct hypothesis testing procedure can attribute values to parameters that allow the prediction to occur, introducing spatial continuity of high or low parameter values as required. It is up to the modeler to judge whether the resulting parameter values and continuity patterns are realistic or not. At the same time, the modeler can decide for him/herself whether any extra misfit that is induced between model outputs and the calibration dataset through inclusion of the hypothesized prediction in the model calibration process is sufficient to invalidate the tested hypothesis. Similarly, misfits incurred by model defects can be tolerated according to the modeler's judgment; a stochastic description of this misfit is not required.

Direct hypothesis testing has been demonstrated by Moore et al. (2010) and Siade et al. (2015). Its guarantee of avoidance of false hypothesis rejection (and hence of modeling failure) is superior to that of other methods. This guarantee comes with a numerical cost, however. The model must be calibrated twice. On one of these calibration occasions, the model must simulate both the past and the hypothesized future. As usual, this cost may be reduced through strategic model abstraction. It may also be reduced through randomized Jacobian computation which requires only as many model runs per inversion iteration as the dimensionality of the solution space; see Doherty (2019) for details.

## Summary

Table 1 attempts to provide a partial summary of the previous few sections. It stresses the importance of tailoring model design, history matching (if the latter is undertaken at all) and uncertainty analysis to the imperatives of decision support.

**Table 1:** Approaches to Quantification and Reduction of the Uncertainties of Decision‐Critical Model Predictions

Dominant source of prediction‐relevant information	Prediction uncertainty quantification and reduction best achieved through…	Model complexity should be sufficient to…	Pitfalls
Expert knowledge and site characterization	Monte Carlo analysis using parameter fields based on geostatistical characterization Worst case analysis	Represent stochasticity of prediction‐salient parameters/processes (or conservative surrogates thereof)	Stochastic geostatistical characterization fails to include prediction‐salient features
Historical system states and fluxes	History matching	Obtain a good fit with the calibration dataset	Relatively straightforward
Both of the above	Linear first‐order second moment analysis Monte Carlo analysis using parameter fields constrained by history matching Direct predictive hypothesis testing	Both of the above	Calibration‐adjustable parameter fields exclude connected (im)permeability History matching may induce predictive bias Hampered by long model run times and numerical instability

## Conclusions

The authors of this paper have attempted to introduce concepts into the discourse on groundwater modeling that can raise its ability to support environmental management. In particular, we have suggested common‐sense metrics for decision‐support modeling, and have discussed ways in which these metrics can be applied. In doing so, we have acknowledged the spectrum of data/prediction contexts in which decision‐support groundwater modeling must take place, and have asserted that most of this type of modeling must inhabit the central portion of this spectrum wherein the values of model predictions must be simultaneously informed by historical measurements of system state and by expert knowledge. We have also suggested that a new modeling mindset is warranted in which models are viewed as receptacles for decision‐pertinent information rather than as simulators of reality. We have challenged the commonly held belief that more complex models are better models, and have stressed the need for strategic abstraction and prediction specificity as a means of providing effective decision support.

To summarize:

- Every numerical model is compromised in its ability to simulate environmental processes.
- Assimilation of decision‐pertinent data is an integral part of decision support modeling.
- Because of data scarcity, post‐assimilation parameter and predictive uncertainties are likely to be significant.
- Abstractions and simplifications should not be viewed as model deficiencies. Rather they should be seen as strategic design features through which the decision support role of modeling is enabled.
- Generally, this enablement must be prediction‐specific.

In light of the above conclusions, we question the widespread practice of viewing a model as a product that, once delivered, can be used to make many predictions of management interest. Instead, we suggest that modeling should be viewed as a process rather than as a product, and that this process should be designed to implement the scientific method. A model then becomes a scientific instrument that is tuned to testing a decision‐specific hypothesis.

Of course, part of the art of decision support modeling must be the framing of hypotheses that models are built to test. A hypothesis can be as simple as the occurrence of a specific unwanted event such as excessive stream depletion or failure to effect contaminant containment. Or it can be more subtle than this. For example, we may hypothesize that management plan B is superior (according to some metric) to management plan A when we are on the threshold of implementing management plan A.

Because the goals of decision support modeling are best served when a modeling strategy is tuned to the testing of a particular hypothesis, multiple models may be required to test multiple hypotheses. In the authors' opinion, this is unlikely to lead to higher modeling costs. Strategic abstractions that enable prediction‐pertinent data assimilation and uncertainty quantification are likely to promulgate faster‐running, numerically more robust models than those which are presently used as a basis for decision support. We submit that construction of a suite of strategically simplified models that process the same data in prediction‐optimal ways will incur no greater cost than the construction and calibration of a giant model that advertises itself as being capable of making all management‐pertinent predictions, and of quantifying all of their uncertainties. We have pointed out that the human cost of building such a fantasy model is as great as its numerical cost, as human intuition drowns in a plethora of numerical details that ultimately have little bearing on environmental management, and that impede rather than enhance the decision support role that such a model is meant to provide.

This paper has discussed the fundamental role played by data assimilation in decision support modeling. The authors have pointed out that decision support groundwater modeling occupies a particularly difficult place in the data assimilation spectrum. Geological media are complex beyond measure or geostatistical characterization. Their hydraulic properties are heterogeneous at every scale. Unwanted management outcomes may arise from connected permeability whose dimensions and locations are unknown, but that may nevertheless be compatible with current geological understandings. Data are scarce, noisy and information‐poor. Information from different sources must be blended. Decision support modeling in this difficult context requires a flexible parameterization scheme that can respond to information gleaned from the historical behavior of the system. At the same time, it must be possible to set limits on parameter variability and spatial connectivity based on expert knowledge, notwithstanding the often abstract connection between a model's parameters and geological reality. Pitfalls abound; if these limits are set too tightly a hypothesized prediction may be falsely rejected; if they are set too loosely, modeling descends into uselessness.

There are some decision support circumstances, however, for which the difficulties associated with decision support modeling are lighter than others. These circumstances should be recognized so that they can be taken advantage of. Where a prediction is only mildly constrained by information contained within a calibration dataset, a modeler has license to dispense with history matching altogether. Without the need to impose history‐matching constraints on parameters, a modeler is free to assimilate expert geological knowledge through the deployment of modern‐day geostatistical methods. The prior probability distribution of a prediction can then be used as a surrogate for its posterior probability distribution. In taking this approach, a modeler should ensure, however, that this prior distribution is not contradicted by measurements of historical system behavior, while not actually calibrating his/her model to these measurements. Alternatively, this situation may allow a modeler to explore a worst‐case scenario using a simple representation of the system under study encapsulated in a fast‐running model.

Another favorable situation is that in which predictions that are required of future system behavior are very similar in nature to observations of past system behavior, and where measurements of the latter abound. Under these circumstances, decision‐pertinent predictions are likely to be solution space dependent. A model that is built to make these predictions need only be as complicated as is required to replicate the measured past.

There can be no simple formula for decision support modeling. All sites are different. Each site is characterized by its own specific data. Nevertheless, some concepts are applicable to all of them. The first of these is the imperative that the scientific method be implemented. The second is acknowledgment that its implementation requires data assimilation. The third is that the specter of failure—whereby modeling generates a false sense of security that unwanted events can be avoided—is an ever‐present consequence of ignoring the first two.
